# Nomogram for predicting the prognosis of metastatic colorectal cancer patients treated with anti-PD1 therapy based on serum lipids analysis

**DOI:** 10.1007/s00262-023-03519-y

**Published:** 2023-08-17

**Authors:** Bijing Xiao, Hui Ouyang, Haimiti Gulizeba, Haiyan Fu, Zhiqiang Wang, Yan Huang

**Affiliations:** Medical Oncology Department, Sun Yat-Sen University Cancer Center, State Key Laboratory of Oncology in South China, Collaborative Innovation Center for Cancer Medicine, No. 651 Dongfeng East Road, Guangzhou, Guangdong 510060 People’s Republic of China

**Keywords:** Metastatic colorectal cancer (mCRC), Anti-PD1 therapy, High-density lipoprotein (HDL-C), Overall survival (OS), Nomogram

## Abstract

**Background:**

Serum lipids have been identified to be used as prognostic biomarkers in several types of cancer. The primary objective of this study was to evaluate the prognostic value of serum lipids in metastatic colorectal cancer (mCRC) patients received anti-PD-1 therapy.

**Methods:**

Pretreatment and the alteration of serum lipids, including apolipoprotein B (ApoB), apolipoprotein A-I (ApoA-I), cholesterol (CHO), high-density lipoprotein cholesterol (HDL-C), low-density lipoprotein cholesterol (LDL-C) and triglyceride (TG) after 2 courses of anti-PD1 therapy, were collected. Kaplan–Meier survival and cox regression analysis were performed to identify the prognostic values on overall survival (OS). Finally, those significant predictors from multivariate analysis were used to construct a nomogram for the prediction of prognosis.

**Results:**

Baseline ApoB, CHO, HDL-C, LDL-C and early changes of ApoB, ApoA-I, HDL-C were statistically significant in the ROC analysis, showing good discriminatory ability in terms of OS. In multivariate analysis, treatment lines, lung metastasis, baseline HDL-C (low vs. high, HR, 6.30; 95% CI 1.82–21.80; *P* = 0.004) and early changes in HDL-C (reduction vs. elevation, HR, 4.59, 95% CI 1.20–17.63; *P* = 0.026) independently predicted OS. The area under the time-dependent ROC curve at 1 year, 2 years and 3 years consistently demonstrated the satisfactory accuracy and predictive value of the nomogram (AUC: 0.88, 0.85, 0.84).

**Conclusion:**

Overall, high level at baseline and an early elevation of HDL-C are correlated with better outcomes in mCRC patients treated with anti-PD1 therapy. The constructed nomogram indicated that the factors are strong predictive markers for response and prognosis to anti-PD-1 therapy in metastatic colorectal cancer.

**Supplementary Information:**

The online version contains supplementary material available at 10.1007/s00262-023-03519-y.

## Introduction

Colorectal cancer (CRC) is the third most frequent malignancy in both men and women worldwide [1] and ranks second in terms of mortality, causing 880,000 deaths in 2018 [2]**.** Programmed death 1 (PD-1) blockade has clinical benefit in microsatellite-instability-high (MSI-H) or mismatch-repair-deficient (dMMR) tumors after previous therapy [3–5]. The single drug anti-PD1 in the NCCN guidelines is a first-line treatment for MSI-H/dMMR patients, and the treatment plan of immunotherapy combined with antiangiogenic drugs is also an important posterior therapy for microsatellite stability (MSS) CRC patients, the median progression survival period is more than 6 months [6]. It is important to note that the efficacy of immunotherapy varies from person to person. Practical and reliable prognostic predictors are desperately needed to differentiate metastatic colorectal cancer (mCRC) patients who are most likely to benefit from PD-1 inhibitors.

Currently, increasing evidence has shown that serum lipids are also correlated with the prevalence and behaviors of various tumors, especially those of the digestive system, including GC, pancreatic cancer, and colorectal cancer [7–9]. Furthermore, studies have also demonstrated that serum lipids may be a promising marker for predicting the efficacy of ICI therapy in solid tumors, including non-small cell lung cancer [10, 11], melanoma and renal cell carcinoma [12]. It has been demonstrated in mouse models that chemo- and immunotherapies can be co-loaded into synthetic HDL (sHDL), delivered locally to the tumor, and can be used to improve survival outcomes significantly compared to chemotherapy alone [13]. Yet, it is unknown if baseline and fluctuations of lipid levels in mCRC patients undergoing anti-PD1 therapy can predict prognosis of mCRC.

The aim of our study was to investigate the potential role of baseline and dynamic change of serum lipids levels in the prognosis of mCRC patients treated with anti-PD1 therapy.

## Methods

### Patient selection

157 mCRC patients (89 patients with mCRC at initial diagnosis and 29 patients with postoperative recurrence) who received anti-PD-1 therapy (monotherapy or combined with other therapies) at Sun Yat-sen University Cancer Center between January 2019 and October 2022 were enrolled in this retrospective study. Patients lacking baseline serum lipids, the alteration of lipids data after 2 cycles of anti-PD-1 therapy, or taking antihyperlipidemic medications during immunotherapy were excluded from the present analysis. All the patients in this study were treated with the dosage and treatment interval of drugs according to the guidelines recommended and appropriately adjusted for the basic characteristics. The biopsy tissues or surgical specimens of patients were used to proceed the genetic testing. The study was approved by Sun Yat-sen University Cancer Center Institutional Review Board. Written informed consent for participation was waived by the Institutional Review Board due to the retrospective nature of the study (SL-B2023-128-01).

### Data collection

Clinical data, including age, gender, Eastern Cooperative Oncology Group (ECOG) performance status (PS), smoking status, primary tumor location, distant metastasis and Microsatellite status, were documented. In addition, the following data of serum lipid profiles at different time points (baseline, 2 cycles after anti-PD1 therapy) were also collected: apolipoprotein B (ApoB), apolipoprotein A-I (ApoA-I), total cholesterol (CHO), high-density lipoprotein cholesterol (HDL-C), low-density lipoprotein cholesterol (LDL-C) and triglyceride (TG).

### Outcomes

Tumor assessment was regularly conducted by CT/MR scans after every 2 cycles of treatment or every 2 months after the completion of therapy. Patient response to anti-PD1 therapy was assessed, based on response evaluation criteria in solid tumor version 1.1 (RECIST 1.1). The primary endpoint was overall survival (OS) defined as the time from initiation of anti-PD-1 therapy to death from any causes. Patients who had not progressed or did not die were censored at the time of the last follow up. Objective response rate (ORR) was defined as the proportion of patients with a complete response (CR) or partial response > 6 months. Disease control rate (DCR) was defined as the proportion of patients with a complete or partial response or duration of stable disease (SD) ≥ 6 weeks.

### Statistical analysis

Receiver operating characteristic (ROC) curve analysis was performed to analyze the area under the ROC curve (AUC), and the Youden index was used to identify the optimal cut-off values for baseline and the alteration of serum lipids. Based on the cut-off value, patients were divided into different groups for further analysis. The correlation of baseline serum lipids and clinical variables was analyzed using the Pearson correlation. Paired-sample t-tests were performed to the comparison of the baseline serum lipids levels and lipids alteration levels after anti-PD1 therapy. Survival analysis, determining the association between serum lipids and overall survival (OS), was conducted using the Kaplan–Meier method and the differences were compared using the log-rank test. Association between baseline characteristics and treatment outcomes was investigated using univariate and multivariate Cox proportional hazards regression analysis. A two-sided *P* value of < 0.05 was used to define the statistical significance. All statistical analyses were conducted using SPSS 25 (IBM, Armonk, NY, USA) and R version 3.3.3. (R Foundation for Statistical Computing, Vienna, Austria).

### Nomogram establishing

Those significant predictors observed in multivariate Cox model (*P* < 0.05) were determined as the independently prognostic factors and then used to construct a nomogram. Time-dependent receiver operating characteristic (ROC) curves at 1-, 2-, 3-year OS were performed, and the area under the curve (AUC) was calculated to evaluate the predictive value of the nomogram. The prediction probability and the observed result frequency were compared through the calibration curve. Based on the nomogram, we calculated each patient’s sum points and find out a best cut-off using the “surv_cutpoint” function in the “survminer” R package, patients was the stratified the risk into low and high group.

## Results

### Patient characteristics at baseline

Of the 157 mCRC patients who treated with anti-PD1 therapy (monotherapy or combined with other therapies; anti-PD-1 including Pembrolizumab, Tirelizumab, Nivolumab, Camrelizumab and Teripulimab) in Sun Yat-sen University Cancer Center between January 2019 and October 2022, a total of 108 patients met eligibility and were then included in the present study. Patient ages at the anti-PD1 therapy initiation ranged from 24 to 85 years (median 59 years). Among the 108 patients included in the study, 65 (60.2%) were males, 60 (55.6%) had ECOG PS of 0, 78 (72.2%) had lung metastasis and 78 (72.2%) had liver metastasis at baseline. Patients who with primary tumors located in colon accounted for 86.1% (*n* = 93). Regarding the microsatellite status, 67 (62.0%) patients had the status of microsatellite stability (MSS) or microsatellite instability-low (MSI-L), and other 41 (38.0%) patients were microsatellite instability-high (MSI-H). The baseline clinical characteristics of the patients are summarized in Table 1. At the last follow-up (January 8, 2023), 43 (39.8%) patients died, 6 patients lost to follow up and 59 patients remained alive. For the best response, 5 patients (4.6%) achieved CR; 28 (25.9%) achieved PR; 49 (45.4%) achieved SD and 26 (24.1%) had PD. The objective response rate (ORR) was 30.6% (33 of 108 patients), and the disease control rate (DCR) was 75.9%.

**Table 1:** Patient characteristics at baseline

	No. of patients (*N* = 108)	Percentage (%)
*Age,* *years*		
Median (range)	59 (24–85)	
< 59	76	70.4
≥ 59	32	29.6
*Gender*		
Male	65	60.2
Female	43	39.8
*ECOG* *PS*		
0	60	55.6
1	47	43.5
2	1	0.9
*Smoker*		
Yes	22	20.4
No	86	79.6
*Location* *of* *primary* *tumor*		
Colon	93	86.1
Rectum	15	13.9
*Lung* *metastasis*		
Yes	78	72.2
No	30	27.8
*Liver* *metastasis*		
Yes	78	72.2
No	30	27.8
*Microsatellite* *status*		
MSS/MSI-L	67	62.0
MSI-H	41	38.0
*Treatment stage*		
First-line	54	50
Second-line	11	10.2
Third-or-higher-line	43	39.8
*Treatment* *type*		
Single-agent immunotherapy	25	23.1
Immunotherapy + chemotherapy	13	12.0
Immunotherapy + targeted therapy	50	46.3
Immunotherapy + chemotherapy + targeted therapy	20	18.5
*Cardiovascular* *disease*		
Hypertension	11	10.2
Coronary heart disease	2	1.9
*Endocrine* *disease*		
Diabetes	9	8.3

Abbreviations: *ECOG* Eastern Cooperative Oncology Group, *MSS* microsatellite stability, *MSI-L* microsatellite instability-low, *MSI-H* microsatellite instability-high

### Correlation analysis of baseline serum lipids level with clinical characteristics

At baseline, the mean (range) levels of baseline ApoB, ApoA-I, CHO, HDL-C, LDL-C and TG were 1.01 (0.43–1.90) g/L, 1.32 (0.67–2.14) g/L, 5.08 (2.07–8.07) mmol/L, 1.28 (0.52–2.31) mol/L, 3.30 (1.08–6.49) mol/L and 1.26 (0.45–4.07) mmol/L, respectively (Supplementary Table S1). The statistical analysis showed that the baseline ApoB was significantly correlated with age (*R* = − 0.21, *P* = 0.029), ECOG-PS (*R* = 0.26, *P* = 0.008), primary tumor location (*R* = 0.30, *P* = 0.001), Microsatellite status (*R* = − 0.27, *P* = 0.004) and liver metastasis (*R* = 0.28, *P* = 0.004). In addition, baseline ApoA-I (*R* = − 0.37, *P* < 0.001) and HDL-C (*R* = − 0.31, *P* = 0.001) were negatively correlated with gender. Also, baseline CHO and LDL-C levels were correlated with primary tumor location, Microsatellite status and liver metastasis. While baseline TG level was not correlated with clinical characteristics (Supplementary Table S2).

### Optimal cut-offs of serum lipids levels for OS stratification using ROC analysis

**Fig. 1:**
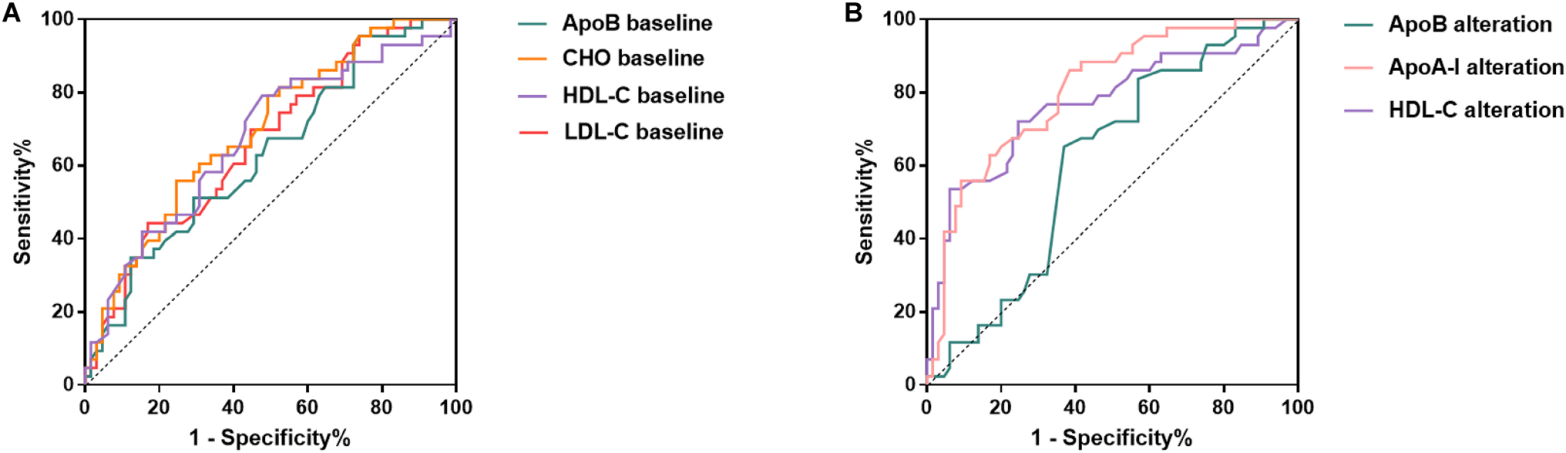
Receiver operating curve analysis of overall survival according to baseline lipids levels (**A**) and the alteration of lipids levels (**B**). ApoB = apolipoprotein B. ApoA-I = apolipoprotein A-I. CHO = cholesterol. HDL-C = high-density lipoprotein cholesterol

Using death from any cause as the end point, ROC analysis was performed to identify the optimal cut-off point of serum lipids levels with the highest sensitivity and specificity. As for baseline lipids levels, results showed that the area under the curve (AUC) for baseline ApoB was 0.631 (*P* = 0.022), 0.694 for CHO (*P* = 0.001), 0.674 for HDL-C (*P* = 0.002) and 0.662 for LDL-C (*P* = 0.005) (Fig. 1A). The optimal cut-off values of baseline lipids were 1.20 g/L for ApoB, 5.30 mmol/L for CHO, 1.19 mmol/L for HDL-C and 3.76 mmol/L for LDL-C, respectively (Supplementary Table S3), while baseline ApoA-I (*P* = 0.223) and TG (*P* = 0.165) were not statistically significant in the ROC analysis.

Moreover, for the alteration of lipids levels after anti-PD1 therapy, AUC for the alteration of ApoB level was 0.613 (*P* = 0.048), based on a -0.005 g/L cut-off; 0.806 for ApoA-I alteration (*P* < 0.0001, cut-off 0.06 g/L); 0.769 for HDL-C alteration (*P* < 0.0001, cut-off − 0.025 mmol/L) (Fig. 1B; Supplementary Table S3). However, the alteration of CHO (*P* = 0.498), LDL-C (*P* = 0.512) and TG (*P* = 0.103) was not statistically significant in the ROC analysis.

### The prognostic values of serum lipids levels at baseline for survival outcomes

Based on the optimal cut-off values using ROC analysis above, patients were separately divided into two groups (low lipids level group and high lipids level group). The association of different groups of baseline lipids levels with treatment outcomes was further analyzed. For the best response, ORR in patients with high levels of ApoB at baseline was 38.5%, which was significantly higher than those with low baseline ApoB (10%) (*P* = 0.004). Similar results had showed that patients with high baseline CHO (ORR: 31.8% vs. 4.2%; *P* = 0.001), HDL-C (ORR: 33.7% vs.0; *P* = 0.028) and LDL-C (ORR: 39.5% vs. 3.7%; *P* < 0.001) had a better ORR that those with low serum lipids at baseline.

**Fig. 2:**
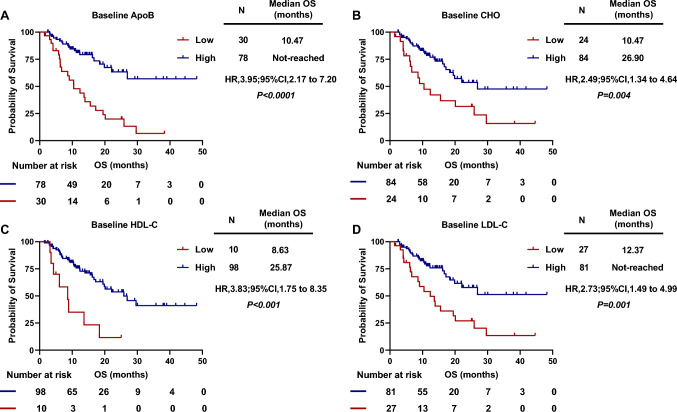
Kaplan–Meier curves for OS. Overall survival according to baseline ApoB (**A**), baseline CHO (**B**), baseline HDL-C (**C**), baseline LDL-C (**D**)

Next, we performed the Kaplan–Meier survival analysis to evaluate the predictive values of serum lipids for PFS. Results showed that patients with low baseline ApoB *(P* < 0.0001), CHO *(P* < 0.0001), HDL-C *(P* = 0.012), LDL-C *(P* < 0.0001) had significantly shorter PFS than patients in the group of high serum lipids (Supplementary Figure S1). Moreover, Kaplan–Meier survival analysis and log-rank test showed that patients with low baseline ApoB had significantly worse OS than those with high baseline ApoB [median OS, 10.47 months vs. not-reached; HR = 3.95, (95% CI 2.17–7.20), *P* < 0.0001; Fig. 2A]. Similar results were observed that patients with low baseline CHO [median OS, 10.47 vs. 26.90 months; HR = 2.49, (95% CI 1.34–4.64), *P* = 0.004; Fig. 2B], low baseline HDL-C [median OS, 8.63 vs. 25.87 months; HR = 3.83, (95% CI 1.75–8.35), *P* < 0.001; Fig. 2C] and low baseline LDL-C [median OS, 12.37 months vs. not-reached; HR = 2.73, (95% CI 1.49–4.99), *P* = 0.001; Fig. 2D] had significantly worse OS.

### The prognostic values of the alteration of serum lipids levels after anti-PD1 therapy for survival outcomes

The mean (range) levels of ApoB, ApoA-I, CHO, HDL-C, LDL-C and TG after 2 cycles of anti-PD1 treatment were 0.97 (0.35–1.90) g/L, 1.36 (0.38–2.22) g/L, 5.01 (1.94–12.05) mmol/L, 1.29 (0.32–2.15) mol/L, 3.18 (1.13–10.18) mol/L and 1.44 (0.51–6.10) mmol/L, respectively (Supplementary Table S1).The mean ± standard deviation (SD) of alteration in the levels of ApoB, ApoA-I, CHO, HDL-C, LDL-C and TG after 2 courses of anti-PD1 therapy (comparing to baseline levels) were − 0.04 ± 0.21 g/L, 0.03 ± 0.31 g/L, − 0.07 ± 1.09 mmol/L, 0.01 ± 0.34 mmol/L, − 0.12 ± 1.00 mmol/L and 0.18 ± 0.72 mmol/L, respectively (Supplementary Table S1). Based on the optimal cut-off values of the alteration of serum lipids levels using ROC analysis, patients were further categorized into two groups (reduction of lipids level group and elevation of lipids level group). ORR in patients with elevation levels of ApoA-I after anti-PD-1 therapy was 43.5%, which was significantly higher than those with reduction of ApoA-I (21.0%) (*P* = 0.012). Also, a better ORR was observed in the group of patients with the elevation of HDL-C (ORR: 41.0% vs. 17.0%; *P* = 0.007). While, there was no statistical difference in ORR between the two groups of ApoB.

**Fig. 3:**
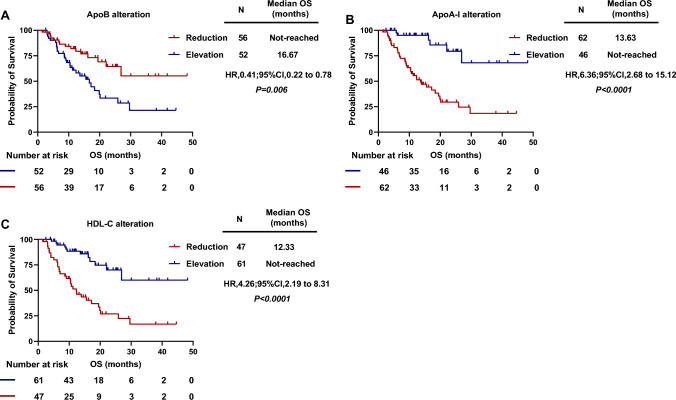
Kaplan–Meier curves for OS. Overall survival according to the alteration of ApoB (**A**) after 2 cycles of anti-PD1 therapy, ApoA-I alteration (**B**), HDL-C alteration (**C**)

Meanwhile, we analyzed the correlation between the alteration of lipids and PFS. Results showed that patients with a reduction of ApoA-I *(P* < 0.0001) and HDL-C *(P* < 0.0001) after anti-PD-1 treatment had significantly shorter PFS than patients with an elevation of serum lipids (Supplementary Figure S2). Also, Kaplan–Meier survival analysis indicated that patients with a reduction of ApoB after two cycles of anti-PD1 treatment had prolonged OS than patients with an elevation of ApoB [HR = 0.41, (95% CI 0.22–0.78), *P* = 0.006; Fig. 3A]. Conversely, patients with a reduction of ApoA-I [HR = 6.36, (95% CI 2.68–15.12), *P* < 0.0001; Fig. 3B], and a reduction of HDL-C [HR = 4.26, (95% CI 2.19–8.13), *P* < 0.0001; Fig. 3C] had a significantly worse OS.

### Univariate and multivariate Cox regression analyses of OS

Furthermore, univariate analysis based on the main characteristics indicated that factors associated with inferior OS included ECOG PS of 1 or 2 (*P* < 0.001), LDH (*P* = 0.024), treatment lines (*P* < 0.001), lung metastasis *(P* < 0.001) and liver metastasis (*P* = 0.006). We further included those significant clinicopathological parameters in univariate analysis into the multivariate model. Multivariate analysis revealed that treatment lines (First line vs. Second line and beyond, HR, 0.33; 95% CI 0.12–0.93; *P* = 0.037) and lung metastasis (yes vs. no, HR, 2.31; 95% CI 1.03–5.14; *P* = 0.041) had the prognostic value for OS. Moreover, as for the baseline and alteration of serum lipids levels, multivariate analysis indicated that baseline low HDL-C (low vs. high, HR, 6.30; 95% CI 1.82–21.80; *P* = 0.004) as well as the reduction of HDL-C (reduction vs. elevation, HR, 4.59, 95% CI 1.20–17.63; *P* = 0.026) after 2 courses of anti-PD1 therapy independently predicted inferior OS (Table 2).

**Table 2:** Predictive factors for OS by univariate and multivariate analysis

		Univariate analyses	Multivariate analyses
		HR(95%CI) *P* value^Φ^	HR(95%CI) *P* value^Φ^
Gender	Male versus female	0.79(0.43–1.45) 0.447	
Age (years)	< 59 versus ≥ 59	1.00(0.52–1.91) 0.991	
ECOG PS	1–2 versus 0	3.72(1.91–7.26) < **0.001**	1.19(0.43–3.26) 0.737
Smoker	Yes versus no	1.22(0.60–2.50) 0.578	
LDH	Low versus high	0.49(0.26–0.91) **0.024**	1.89(0.75–4.74) 0.174
Treatment lines	1st versus ≥ 2nd	0.15(0.07–0.32) < **0.001**	0.33(0.12–0.93) **0.037**
Lung metastasis	Yes versus no	5.49(2.86–10.54) < **0.001**	2.31(1.03–5.14) **0.041**
Liver metastasis	Yes versus no	15.80(2.17–114.77) **0.006**	6.76(0.86–52.97) 0.069
ApoB baseline	Low versus high	3.95(2.17–7.20) < **0.001**	2.26(0.79–6.48) 0.130
CHO baseline	Low versus High	2.49(1.34–4.64) **0.004**	1.57(0.34–7.23) 0.562
HDL-C baseline	Low versus high	3.83(1.75–8.35) < **0.001**	6.30(1.82–21.80) **0.004**
LDL-C baseline	Low versus high	2.73(1.49–4.99) **0.001**	0.33(0.07–1.68) 0.183
ApoB alteration	Reduction versus elevation	0.41(0.22–0.78) **0.006**	1.78(0.64–4.92) 0.268
ApoA-I alteration	Reduction versus elevation	6.36(2.68–15.12) < **0.001**	1.22(0.26–5.67) 0.804
HDL-C alteration	Reduction versus elevation	4.26(2.19–8.13) < **0.001**	4.59(1.20–17.63) **0.026**

Abbreviations: *OS* overall survival, *ECOG* *PS* Eastern Cooperative Oncology Group performance status, *LDH* lactate dehydrogenase, *ApoB* apolipoprotein B, *ApoA-I* apolipoprotein A-I, *CHO* Cholesterol, *HDL-C* high-density lipoprotein cholesterol, *LDL-C* low-density lipoprotein cholesterol, *TG* Triglyceride, *HR* hazard ratio, *CI* Confidence interval

^**Φ**^Values in boldface indicate *P* values < 0.05

### Predictive capacity of a combined baseline HDL-C and the early changes of HDL-C

**Fig. 4:**
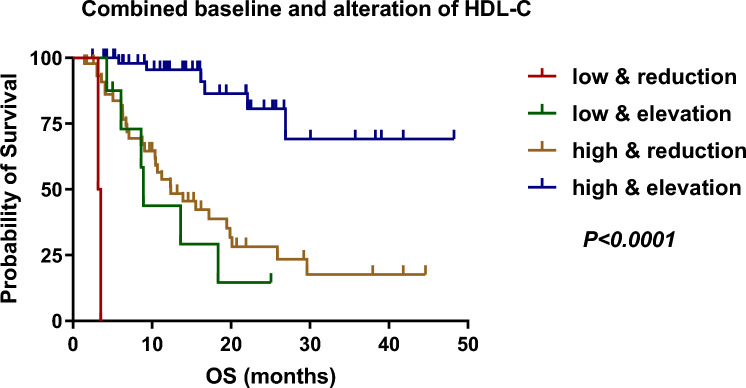
Kaplan–Meier survival curves for OS according to the combination with baseline HDL-C and the alteration of HDL-C

For additional verification, we further stratified patients into four groups (baseline HDL-C low/high and the alteration of HDL-C reduction/elevation). The combination of both prognostic factors improved risk stratification for OS (*P* < 0.0001). Kaplan–Meier survival analysis showed that patients with both baseline low HDL-C as well as a reduction of HDL-C had the worst survival outcome (*n* = 2, median OS = 3.40 months). Patients who had either adverse prognostic feature (baseline low HDL-C or early reduction of HDL-C) had intermediate survival. As expected, we found that patients with both baseline high HDL-C and an elevation of HDL-C after anti-PD1 therapy (*n* = 53) had the best survival outcomes, with a not-reached median OS (Fig. 4), further verifying the prognostic value of HDL-C.

### Prognostic nomogram for prediction of OS

**Fig. 5:**
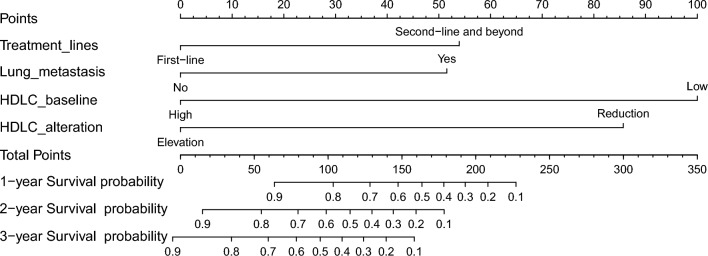
Nomogram for predicting the probability of 1-, 2- and 3-year OS in mCRC patients after anti-PD1 therapy

**Fig. 6:**
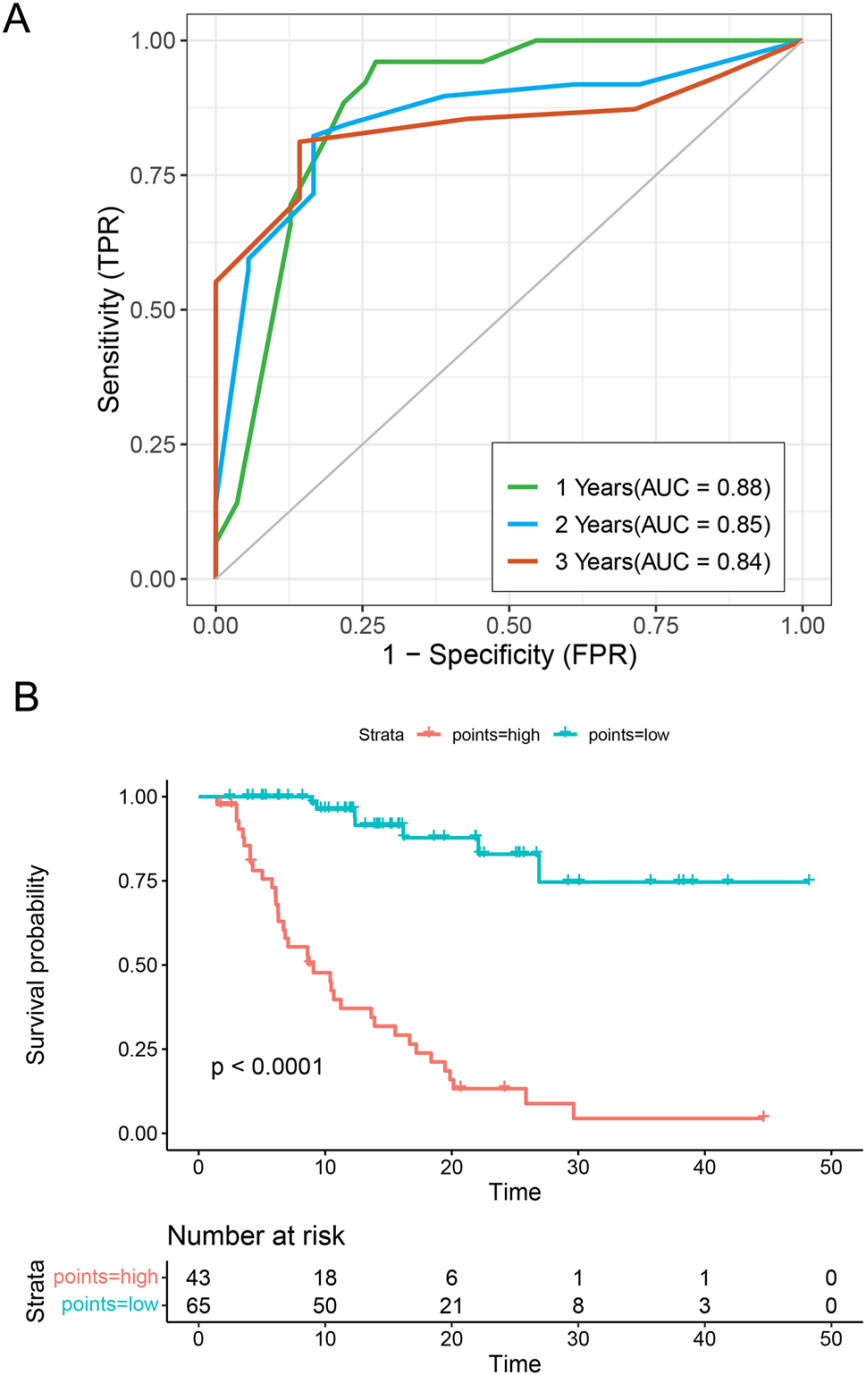
**A** The prognostic accuracy of the nomogram was verified by using time-dependent ROC curves and AUCs at 1 year, 2 years, and 3 years. **B** Kaplan–Meier curve for OS with risk stratification

The significant variables from the multivariate Cox analysis, including treatment lines, lung metastasis, baseline HDL-C and alteration of HDL-C were used to establish a prognostic nomogram for OS. Each factor corresponds to a specific point by drawing a line straight up to the score axis, and the total points were calculated by adding up the scores of all the factors. The probability of survival was demonstrated by making a vertical line from the total score axis to intersect the survival probability axis of 1, 2, and 3 years (Fig. 5). The area under the time-dependent ROC curve at 1 year, 2 years and 3 years consistently demonstrated the satisfactory accuracy and predictive value of the nomogram (AUC: 0.88, 0.85, 0.84) (Fig. 6A). The calibration plot showed satisfactory consistency between the nomogram-predicted OS and actual survival outcomes (Supplementary Figure S3). Based on the constructed nomogram, 100 was the best cutoff value for the total points (Supplementary Figure S4). All the patients were then divided into high-risk group (> 100) and low-risk group (≤ 100), based on the cutoff value. A remarkable difference in OS was observed that patients at the high-risk group had significantly poorer survival outcomes than patients at the low-risk group (*P* < 0.0001) (Fig. 6B).

## Discussion

Approximately 25% of colorectal cancer patients are found to be in stage IV, and another 25–50% of early-stage patients go on to develop metastatic disease [14–17]. Patients with advanced stage IV had much worse prognoses than those with early stage, and the 5-year survival rate of stage IV disease drops to about 10% [18, 19]. Nevertheless, serum lipids are increasingly recognized to play an important role in the tumor initiation and progression, which had been supported as predictive and prognostic markers for cancer patients receiving ICI therapy. A better understanding of the association of CRC and lipids will not only give insight into its pathogenesis, but is also important for the development of novel biomarkers and therapeutic strategies.

It is widely known that the accumulation of excess cholesterol is a general characteristic of tumor tissues [20]. Furthermore, tumor cells are equipped to weaken inflammatory activity of T cells and macrophages by promoting free cholesterol efflux, resulting in an increase in the proportion of T regulatory cells (Tregs) [21]. Previous studies have considered that the failure of anti-tumor immunity is caused by energy metabolism [22, 23]. It’s acknowledged that in the tumor microenvironment (TME) immune cells compete fiercely for proliferation due to an inadequate vascular exchange by limiting nutrients [24]. As essential fuel and metabolic components, lipids could allow anti-tumor immune cells to survive in a harsh TME established by cancer cells. Therefore, the elevation of serum lipids may compensate for the lipid deficiency in immune cells in the tumor microenvironment. Previous studies have demonstrated that lipid-deficient immune cell activity in the tumor microenvironment can potentially be restored by the addition of exogenous lipids, thereby enhancing antitumor activity [25, 26].

Based on these findings, we sought to explore the role of serum lipids in the prognosis of mCRC patients with anti-PD-1-based therapy. Our results indicated that patients with high level of HDL-C at baseline had a prolonged overall survival than those with low level of HDL-C. Meanwhile, patients with an increased HDL-C after 2 cycles of anti-PD-1 treatment had more favorable outcomes, demonstrating the prognostic value of HDL-C in mCRC patients undergoing immunotherapy. HDL-C accounts for about 20% of total plasma cholesterol, and it was nicknamed the “Vascular Wall Cleaner”. Several studies have shown a negative association between HDL-C and cancer incidence [27, 28], with the inclusion of colorectal cancer [29]. A significant negative correlation between HDL and Treg levels has been observed in humans by extensively observing changes in HDL-C levels and lipoprotein composition in immune diseases [30, 31], Moreover, research shows that HDL can influence the activity of monocyte/macrophages, DCs, and lymphocytes mainly by modulating cholesterol content in lipid rafts and receptor activity, as well as by influencing immune cell activation.

However, this study has some limitations. First, due to the retrospective design of the study, the number of patients was limited and conducting research only in one center. Further prospective studies are needed to confirm our findings. Secondly, some of patients in this cohort received combination treatment, confounding factors were inevasible. Finally, HDL-C may be affected by multiple factors, such as dietary structure, steroid therapy or other stress triggers, which were not evaluated in this study.

## Conclusion

In conclusion, our findings demonstrated that a high level of HDL-C at baseline or an increased HDL-C after 2 cycles of treatment predict better survival outcome of mCRC patients undergoing anti-PD1 therapy. Our study constructed a nomogram based on the treatment lines, lung metastasis, baseline HDL-C and the alteration of HDL-C after immunotherapy, showing that the factors are strong predictive markers for response and prognosis to anti PD-1 therapy in metastatic colorectal cancer. This parameter may serve as a novel effective marker and therefore assisting in treatment regimen selection.

### Supplementary Information

Below is the link to the electronic supplementary material.

Supplementary file1 (PDF 650 KB)

## Data Availability

The datasets generated during and/or analyzed during the current study are available from the corresponding author on reasonable request.
