# Supplementary material for: Nomogram for predicting the prognosis of metastatic colorectal cancer patients treated with anti-PD1 therapy based on serum lipids analysis
Source: Cancer Immunol Immunother. 2023 Aug 17;72(11):3683–92. doi: 10.1007/s00262-023-03519-y (PMC10576722; doi:10.1007/s00262-023-03519-y)
Supplement: Supplementary file 1 — Supplementary file1 (PDF 650 KB) [file 262_2023_3519_MOESM1_ESM.pdf]

**Lipid**	**Mean ± SD**	**Pvalue**[ϕ]
**ApoB (g/L)**		0.729
Baseline	1.01±0.26	
After anti-PD1 therapy	0.97±0.29	
Difference[a]	-0.04±0.21	
**ApoA-I (g/L)**		0.541
Baseline	1.32±0.30	
After anti-PD1 therapy	1.36±0.35	
Difference[a]	0.03±0.31	
**CHO (mmol/L)**		0.646
Baseline	5.08±1.12	
After anti-PD1 therapy	5.01±1.41	
Difference[a]	-0.07±1.09	
**HDL-C (mmol/L)**		0.616
Baseline	1.28±0.38	
After anti-PD1 therapy	1.29±0.39	
Difference[a]	0.01±0.34	
**LDL-C (mmol/L)**		0.651
Baseline	3.30±1.05	
After anti-PD1 therapy	3.18±1.28	
Difference[a]	-0.12±1.00	
**TG (mmol/L)**		0.551
Baseline	1.26±0.64	
After anti-PD1 therapy	1.44±0.84	
Difference[a]	0.18±0.72	

Supplementary Table S1. Baseline and the alteration of lipids levels

SD=standard deviations

ApoB=Apolipoprotein B

ApoA-I= apolipoprotein A-I

CHO=Cholesterol

HDL-C=High-Density Lipoprotein Cholesterol

LDL-C=Low-Density Lipoprotein Cholesterol

TG=Triglyceride

aDifference = baseline lipids –after anti-PD1 therapy lipids.

ϕCompared with paired t-test.

Variables		ApoB		ApoA-I		CHO		HDL-C		LDL-C		TG	
		**R**	***P* value**	**R**	***P* value**	**R**	***P* value**	**R**	***P* value**	**R**	***P* value**	**R**	***P* value**
Age	≤59 vs. >59	**-0.21**	**0.029**	0.04	0.722	-0.15	0.118	-0.10	0.283	-0.15	0.114	0.08	0.408
Gender	Male vs. Female	-0.05	0.534	**-0.37**	**<0.001**	-0.12	0.220	**-0.31**	**0.001**	-0.08	0.938	-0.05	0.577
Smokers	Yes vs. No	0.03	0.771	-0.17	0.078	-0.09	0.354	-0.17	0.087	-0.04	0.684	0.08	0.404
ECOG PS	0 vs. 1-2	**0.26**	**0.008**	0.07	0.473	**0.25**	**0.009**	0.16	0.093	0.18	0.056	0.16	0.102
Primary tumor location	colon vs. rectum	**0.30**	**0.001**	-0.06	0.553	**0.25**	**0.009**	0.05	0.602	**0.25**	**0.010**	-0.04	0.659
Microsatellite status	MSS/MSI-L vs.MSI-H	**-0.27**	**0.004**	-0.10	0.292	**-0.33**	**0.001**	-0.23	0.016	**-0.27**	**0.005**	0.02	0.864
Liver Metastasis	Yes vs. No	**0.28**	**0.004**	-0.07	0.480	**0.30**	**0.001**	0.10	0.315	**0.28**	**0.004**	-0.05	0.646

Supplementary Table S2 Correlation Analysis of Serum Lipids levels with clinical characteristics[Φ]

**Lipid**	**Baseline**	**Alteration**
ApoB (g/L)	1.20	-0.005
ApoA-I (g/L)		0.06
CHO (mmol/L)	5.30	
HDL-C (mmol/L)	1.19	-0.025
LDL-C (mmol/L)	3.76	

Supplementary Table S3 Optimal cut-off of serum lipids

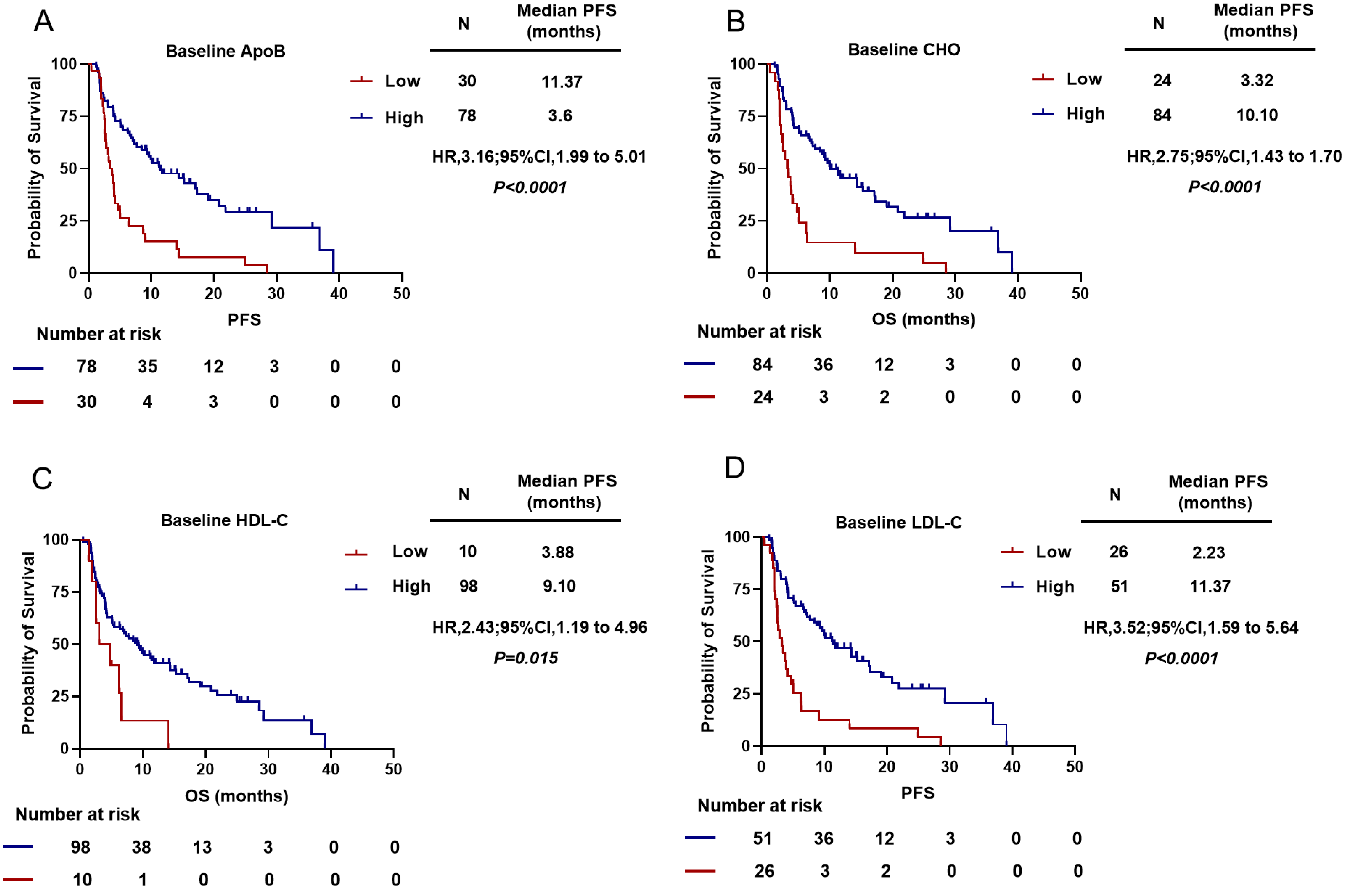

Supplementary Figure S1. Kaplan-Meier curves for PFS. PFS according to baseline ApoB(A), baseline CHO (B), baseline HDL-C(C), baseline LDL-C(D).

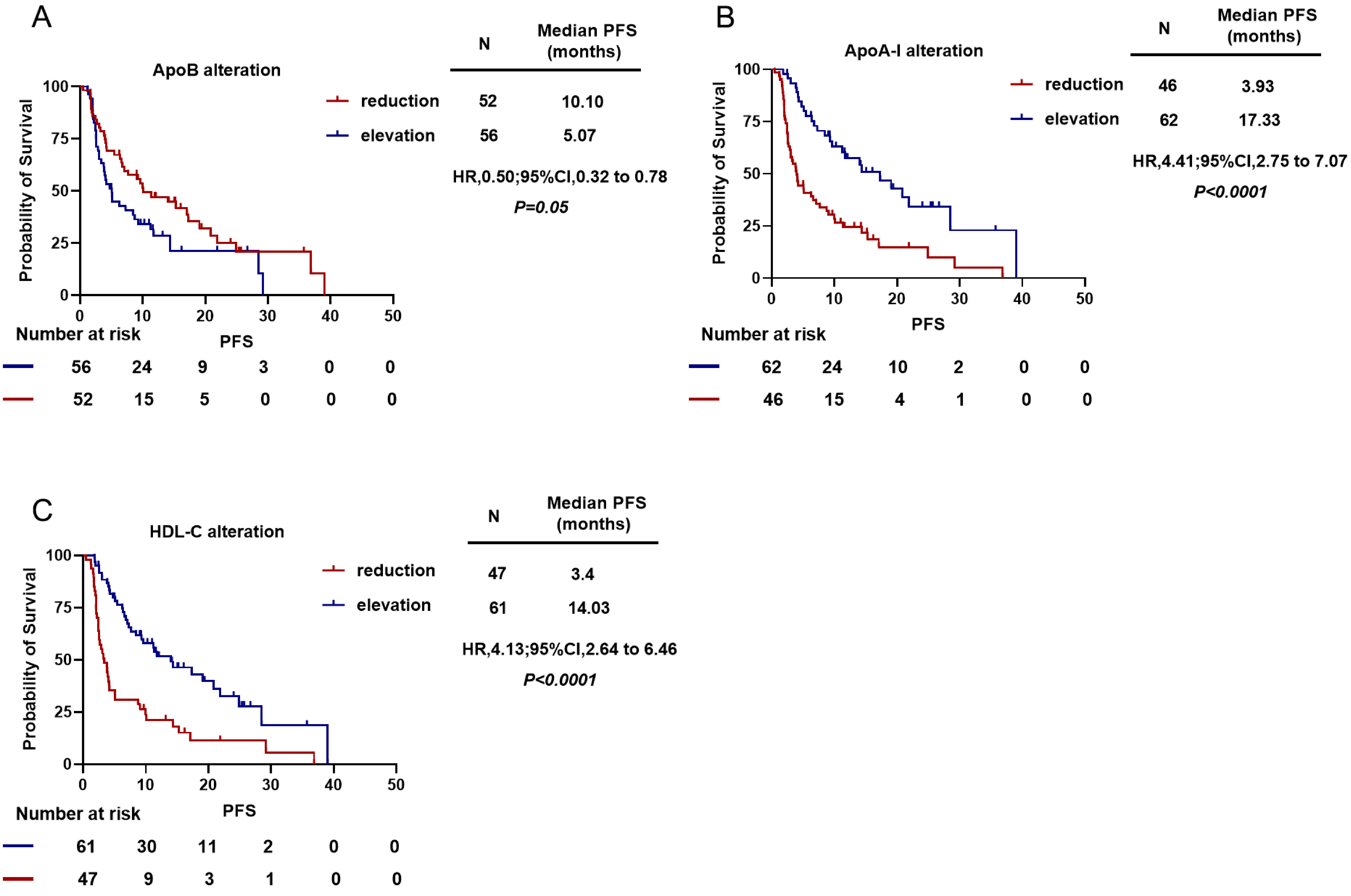

Supplementary Figure S2. Kaplan-Meier curves for PFS. PFS according to the alteration of ApoB(A) after 2 cycles of anti-PD1 therapy, ApoA-I alteration(B), HDL-C alteration (C).

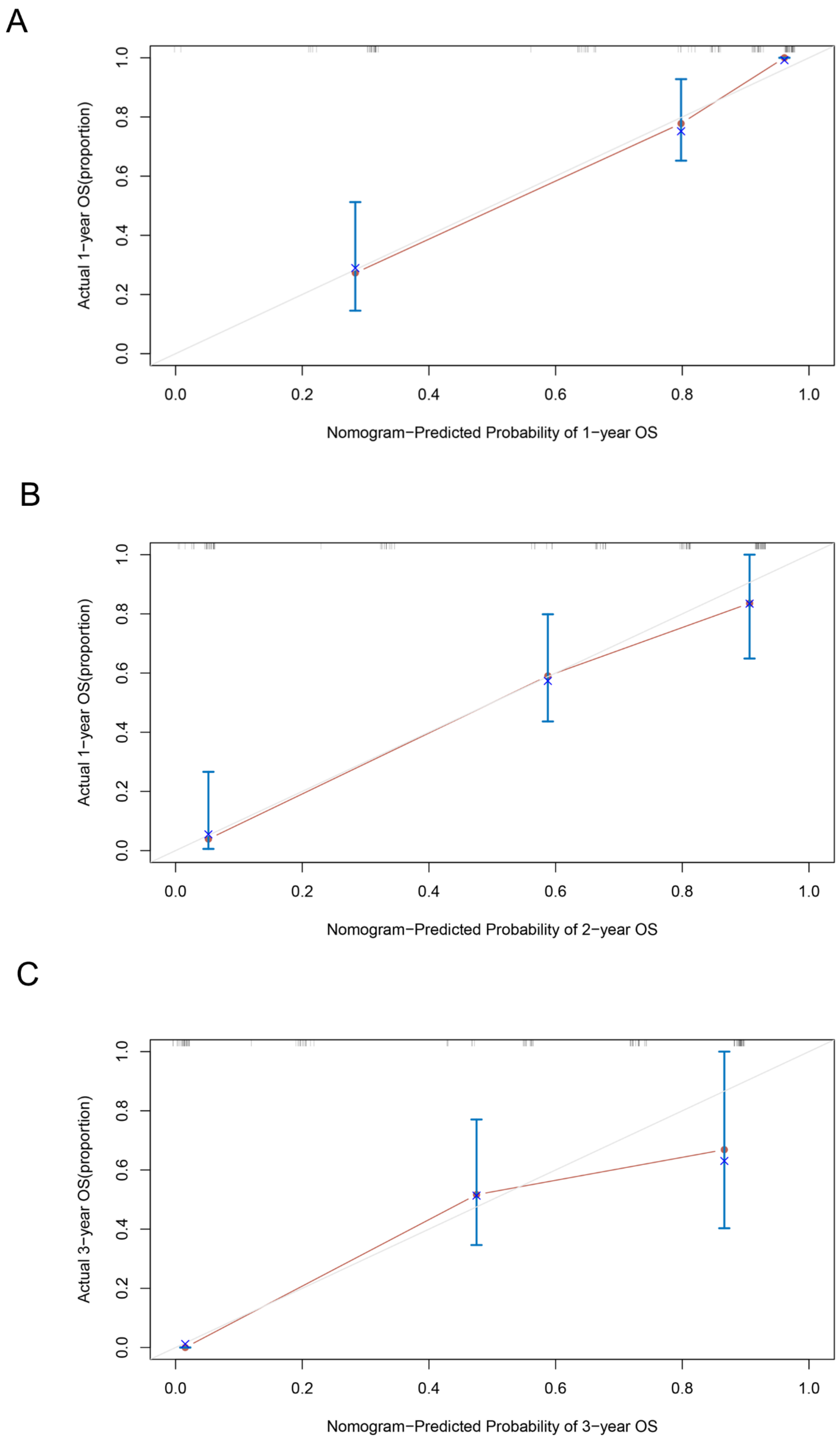

Supplementary Figure S3. The calibration curve for predicting the 1-year (A), 2-year (B) and 3-year (C) OS of patients.

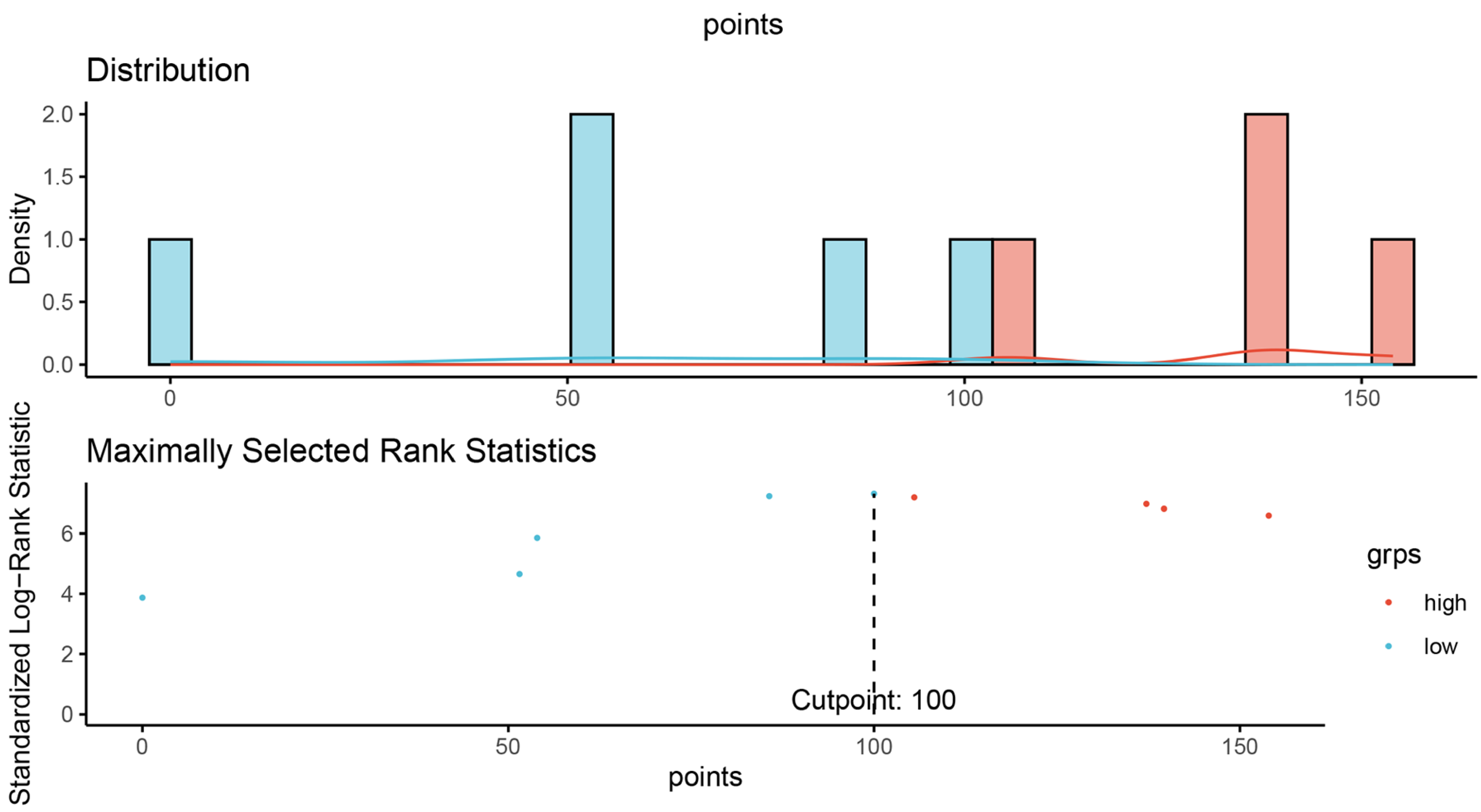

Supplementary Figure S4. The optimal cut-off value determined by the total points was 100.
